# Tackling intraspecific genetic structure in distribution models better reflects species geographical range

**DOI:** 10.1002/ece3.2010

**Published:** 2016-02-26

**Authors:** Arnald Marcer, Belén Méndez‐Vigo, Carlos Alonso‐Blanco, F. Xavier Picó

**Affiliations:** ^1^ CREAFCerdanyola del Vallès08193Spain; ^2^ Univ Autònoma de BarcelonaCerdanyola del Vallès08193Spain; ^3^ Departamento de Genética Molecular de PlantasCentro Nacional de Biotecnología (CNB)Consejo Superior de Investigaciones Científicas (CSIC)28049MadridSpain; ^4^ Departamento de Ecología IntegrativaEstación Biológica de Doñana (EBD)Consejo Superior de Investigaciones Científicas (CSIC)41092SevillaSpain

**Keywords:** *Arabidopsis thaliana*, genetic structure, global climate change, potential distribution range, species distribution models

## Abstract

Genetic diversity provides insight into heterogeneous demographic and adaptive history across organisms’ distribution ranges. For this reason, decomposing single species into genetic units may represent a powerful tool to better understand biogeographical patterns as well as improve predictions of the effects of GCC (global climate change) on biodiversity loss. Using 279 georeferenced Iberian accessions, we used classes of three intraspecific genetic units of the annual plant *Arabidopsis thaliana* obtained from the genetic analyses of nuclear SNPs (single nucleotide polymorphisms), chloroplast SNPs, and the vernalization requirement for flowering. We used SDM (species distribution models), including climate, vegetation, and soil data, at the whole‐species and genetic‐unit levels. We compared model outputs for present environmental conditions and with a particularly severe GCC scenario. SDM accuracy was high for genetic units with smaller distribution ranges. Kernel density plots identified the environmental variables underpinning potential distribution ranges of genetic units. Combinations of environmental variables accounted for potential distribution ranges of genetic units, which shrank dramatically with GCC at almost all levels. Only two genetic clusters increased their potential distribution ranges with GCC. The application of SDM to intraspecific genetic units provides a detailed picture on the biogeographical patterns of distinct genetic groups based on different genetic criteria. Our approach also allowed us to pinpoint the genetic changes, in terms of genetic background and physiological requirements for flowering, that Iberian *A. thaliana* may experience with a GCC scenario applying SDM to intraspecific genetic units.

## Introduction

Classical taxonomic designations (e.g., species) may not represent the ecological and evolutionary units that matter most to understand the mechanisms that shape biogeographical patterns. Despite its elusiveness, a species can be described as an assemblage of genetic lineages varying in their genetic inter‐relationship and spatial distribution. Such intraspecific genetic structure, whatever its extent, is accounted for by populational processes that determine allelic frequencies (i.e., mutation, migration, random drift, or selection) and long‐term demographic fate. Hence, tackling intraspecific genetic variation is unavoidable if we aim to comprehend key topics in biological sciences including evolution, biogeography, conservation biology, and species response to climate change (Benito‐Garzón et al. 2011). However, there is no denying that working at the intraspecific genetic level poses several challenges to researchers because of the need to work with large sample sizes at wide geographical ranges.

The value of intraspecific genetic variation has recently gained great relevance in a context of GCC (global climate change) (Sork et al. 2010; Bálint et al. 2011; Beatty and Provan 2011; Collevatti et al. 2011; Habel et al. 2011; Hoffmann and Sgrò 2011; Alsos et al. 2012; Espíndola et al. 2012; Pfenninger et al. 2012; Rubidge et al. 2012; Pauls et al. 2013; Assis et al. 2014; Yannic et al. 2014; Gotelli and Stanton‐Geddes 2015). Given the substantial rate of GCC predicted for the current century (IPCC 2013), many species may not be able to keep pace with predicted climatic conditions. They will experience substantial shifts in their geographical patterns and a generalized impoverishment of their genetic diversity (Pauls et al. 2013), which is the basis for any adaptive change to new environmental conditions (Jump et al. 2009; Fournier‐Level et al. 2011). The main cause of such a loss of genetic diversity has to do with dramatic changes in the spatio‐temporal distribution of genetic variants eventually affecting the organisms’ adaptive potential (Hoffmann and Sgrò 2011; Pauls et al. 2013; Thuiller et al. 2013).

Considering intraspecific genetic variation in a context of GCC is a methodological rather than a conceptual challenge. In other words, it is difficult to define the genetic level (e.g., polymorphism, haplotype, genetic cluster) to use, which in turn determines the sampling effort required for obtaining genetic data of interest (Pfenninger et al. 2012; Gotelli and Stanton‐Geddes 2015). Regardless of the intraspecific genetic level, it is difficult to generalize about the effects of GCC beyond the expected relationship between range contractions and loss of genetic diversity. In this context, there are notable examples of studies dealing with intraspecific genetic variation (Thomassen et al. 2010a; Benito‐Garzón et al. 2011; Jay et al. 2012). However, we clearly need to increase their number to better interpret the effects of GCC on organisms’ geographical shifts and subsequent gain/loss of genetic diversity, especially studies including extensive sampling and intraspecific genetic variation from various sources.

In this study, we combined SDM (species distribution models) with genetic analyses using a collection of 279 populations of the annual plant *Arabidopsis thaliana* from the Iberian Peninsula. *Arabidopsis thaliana* is native to W Eurasia and has been naturalized worldwide (Hoffmann 2002). It is worth emphasizing that the Iberian Peninsula is one of the most diverse regions of the species’ distribution range considering genetic and ecological criteria (Picó et al. 2008; Cao et al. 2011; Weigel 2012). We used various sources of genetic variation that may matter to understand *A. thaliana*'s biogeographical patterns in the Iberian Peninsula as well as GCC‐induced range fluctuations: nuclear genome‐wide SNPs (single nucleotide polymorphisms), chloroplastic SNPs, and phenotypic variation in the vernalization requirement for flowering time. We have already learned that genetic variation of *A. thaliana* is geographically structured in the Iberian Peninsula because of the multiple Pleistocene refugia contained in the SW part of the species’ range in Eurasia (Picó et al. 2008; Brennan et al. 2014). Furthermore, Iberian *A. thaliana* has been shown to adapt to different altitudes and climatic conditions by adjusting its flowering time to the different Iberian environmental conditions (Méndez‐Vigo et al. 2011, 2013; Manzano‐Piedras et al. 2014). Local adaptation points to genetic differentiation being partly explained by environmental heterogeneity (Thomassen et al. 2010b; Anderson et al. 2011; Fournier‐Level et al. 2011; Weinig et al. 2014) and allows for the use of correlation models to better understand the geographical distribution of species and their intraspecific genetic units.

Here, we decompose *A. thaliana* into consistent genetic units, which capture intraspecific variation from different sources of genetic variation, to better look into the effects of GCC on plant distribution ranges and genetic diversity. The sources of genetic variation used allow the inference of genetic units that capture the heterogeneous demographic and adaptive history of *A. thaliana* across its distribution range in the Iberian Peninsula. In this study, we address the following specific questions. First, how do SDM predict the current distribution of *A. thaliana* and that of its intraspecific genetic units in the Iberian Peninsula? Second, which are the environmental variables that account for the distribution of genetic units? And third, which are the main methodological limitations that have to be addressed to improve our GCC predictions based on intraspecific genetic variation?

## Materials and Methods

### Source data

We used 279 accessions of the plant *Arabidopsis thaliana* (L.) Heyhn. (Brassicaceae) from the Iberian Peninsula (ca. 800 × 700 km^2^; 36.00° N – 43.48° N, 3.19° E – 9.30° W) collected during the period 2004–2009. *Arabidopsis thaliana* is an annual, self‐compatible, and self‐fertile plant exhibiting a persistent seed bank (Montesinos et al. 2009) and different life cycles characterized by winter‐ and spring‐germinated cohorts of individuals (Picó 2012). Study accessions were separated by 1–1042 km and encompassed all habitats and environments where the species occurs in the Iberian Peninsula from seaside to alpine locations (1–2662 m.a.s.l; Picó et al. 2008; Manzano‐Piedras et al. 2014). Accessions represented the most common phenotype, in terms of vernalization requirement for flowering and flowering time, from their respective populations (Manzano‐Piedras et al. 2014).

Nuclear genetic data were obtained from 250 SNPs, which were previously analyzed in this set of 279 *A. thaliana* accessions (Manzano‐Piedras et al. 2014). All accessions were genetically different from each other. These SNPs are located across the whole genome at presumably neutral regions spaced at 0.5Mb average intervals (range = 0.11 kb to 1.82 Mb), including markers that are polymorphic in Central Europe, the Iberian Peninsula, and worldwide. These SNPs exhibit very low ascertainment bias for Iberian *A. thaliana* accessions and they can be analyzed simultaneously as shown elsewhere (Picó et al. 2008; Gomaa et al. 2011; Méndez‐Vigo et al. 2011; Manzano‐Piedras et al. 2014).

The chloroplast genome was analyzed with 15 SNPs with an average distance of 7.6 kb between adjacent SNPs, covering most of the chloroplast genome. Eight of these SNPs were selected from DNA polymorphisms previously described in a worldwide collection of *A. thaliana* accessions (Jakobsson et al. 2007). The remaining SNPs were developed from polymorphisms found by sequencing seven chloroplast DNA fragments (Jakobsson et al. 2007) in a panel of 16 Iberian accessions spanning the specific geographical range of this study. Overall, chloroplast SNPs were genotyped in 181 of 279 *A. thaliana* Iberian accessions (Fig. S1) using the VeraCode method through CEGEN genotyping service (http://www.cegen.org).

For each *A. thaliana* accession, the OVR (obligate vernalization requirement) was quantified by calculating the proportion of nonflowering individuals when grown without a previous vernalization treatment for flowering induction, as previously described (Méndez‐Vigo et al. 2011). We selected this trait because the vegetative‐to‐reproductive transition regulated by low winter temperature has been shown to be a major life‐history trait that *A. thaliana* adapts to the environmental heterogeneity of the Iberian Peninsula (Méndez‐Vigo et al. 2011, 2013; Manzano‐Piedras et al. 2014). In 2012, 4‐day‐old seedlings germinated in Petri dishes from all 279 accessions were planted and grown simultaneously in a plant growth chamber at 21°C with a long‐day photoperiod at the facilities of the Centro Nacional de Biotecnología (CNB‐CSIC) in Madrid. The experimental design included three blocks and six individuals per accession and block (279 accessions × 3 blocks per accession × 6 individuals per block = 5022 individuals). Flowering initiation of each plant was recorded when plants had the first open flower and OVR was quantified when flowering initiation of all accessions ceased and only vegetative plants remained alive. The experiment was terminated after 220 days. Accessions were categorized as OVR if at least 50% of individuals required vernalization for flowering and non‐OVR otherwise. On average, OVR and non‐OVR accessions exhibited 88.9 ± 1.7% (*N *=* *82; range = 50.0–100.0%) and 4.2 ± 0.7% (*N *=* *197; range = 0.0–46.7%) of individuals with obligate vernalization requirement, respectively (Fig. S1).

### Intraspecific genetic units

The genetic relationships among *A. thaliana* accessions based on nuclear SNPs were assessed by Bayesian means using the model‐based algorithm implemented in STRUCTURE v.2.2. (Pritchard et al. 2000) following the protocols described elsewhere (Méndez‐Vigo et al. 2011, 2013). All 279 *A. thaliana* accessions were nonredundant multilocus genotypes (average ± SE genetic distance among accession pairs = 0.33 ± 0.05; range = 0.04–0.49). STRUCTURE inferred four genetic clusters in the Iberian Peninsula (Figs. S1, S2), which was consistent with previous studies (Picó et al. 2008; Méndez‐Vigo et al. 2011; Brennan et al. 2014). Cluster membership coefficients per genetic cluster were (average ± SE): 0.62 ± 0.01 (*N *=* *150), 0.60 ± 0.02 (*N *=* *58), 0.80 ± 0.03 (*N *=* *38), and 0.75 ± 0.03 (*N *=* *33) for genetic clusters C1, C2, C3, and C4, respectively. In this study, we assigned each accession to the genetic cluster whose membership coefficient was equal or higher than 0.5, giving a total of 116, 36, 31, and 29 accessions for genetic clusters C1, C2, C3, and C4, respectively.

All 15 chloroplast SNPs were polymorphic and yielded 14 chloroplast haplotypes, that is, chlorotypes. The relationship among chlorotypes was analyzed using the median‐joining algorithm implemented in NETWORK v.4.6.1.2. (Fluxus Technology Ltd., Suffolk, England). Accessions were classified into three chlorotype groups (A, B, and C) based on the topology of the chlorotype network (Fig. S3). The final number of accessions included in each chlorotype group was 102 for A (6 chlorotypes), 63 for B (5 chlorotypes), and 16 for C (3 chlorotypes), respectively (Fig. S3).

### Distribution modeling

We used MaxEnt v.3.3.3k (Phillips et al. 2006), a presence–background modeling technique based on the maximum entropy principle to model the current potential distribution (Phillips et al. 2006; Jiménez‐Valverde et al. 2008) of *A. thaliana* as species and of each of its genetic units. Finally, we projected the model into a GCC scenario.

We considered three sources of environmental predictors as factors determining the distribution of *A. thaliana* in the Iberian Peninsula: climate, land use, and soil. Environmental layers were extracted from a geographical information system generated for the collection of natural *A. thaliana* populations across the Iberian Peninsula (see Manzano‐Piedras et al. 2014). Climate, land use, and soil variables were obtained from different digital geographical databases publicly available on the Internet: the Digital Climatic Atlas from the Iberian Peninsula (http://opengis.uab.es/wms/iberia/en_index.htm; data accessed on October 15th, 2014), the CORINE Land Cover 2000 (http://www.eea.europa.eu/publications/COR0-landcover; data accessed on October 15th, 2014) and the Soil Geographical Database from Eurasia v.4 (http://esdac.jrc.ec.europa.eu/; data accessed on October 15th, 2014), respectively.

Eight model predictors were chosen from these sources. Of these, five were bioclimatic variables with a degree of collinearity (Spearman's correlation coefficient) lower than 0.7. The 279 *A. thaliana* accessions were climatically characterized by annual mean temperature (BIO1), mean diurnal temperature range (BIO2), temperature seasonality (BIO4), annual precipitation (BIO12), and precipitation seasonality (BIO15). With respect to land use, accessions were characterized by the percentage of agricultural land and the percentage of urbanized area within a circular area (500 m radius) around the GPS coordinate of *A. thaliana* accessions. The rest of the circular area was occupied by woody vegetation. Finally, the soil pH was also assigned to each *A. thaliana* accession.

As our goal was not to compare multiple GCC scenarios but to split a species into genetic units to show their GCC‐driven range fluctuations, we chose the 2070 RCP8.5 (HadGEM2, Met Office Hadley Centre ESM) greenhouse gas concentration scenario that predicts high emissions and surface temperature changes to exceed 2°C by the second half of this century in the Mediterranean (IPCC 2013). We ran two sets of models: (1) models with climatic, land use, and soil variables to model current potential distribution, and (2) models with only the climatic variables in order to be able to project them into future scenarios of GCC (vegetation or soil databases are not available for such scenarios).

We used MaxEnt with its default parameters but took particular attention to several important aspects of SDM such as bias, overfitting, background selection, and spatial autocorrelation. We corrected for bias (Merow et al. 2013) by providing MaxEnt's bias file option a road density layer. In order to minimize overfitting (Radosavljevic and Anderson 2014), we tested model performance with different *β*‐regularization coefficients, the solution offered by MaxEnt to relax model fit, and performed 10‐fold cross‐validation. After checking that there was no model improvement, which means lack of strong bias in occurrence data (Anderson and Gonzalez 2011), we used the default value (*β *= 1) in all simulations. Background selection is also critical in MaxEnt when extrapolating to novel environmental scenarios (Webber et al. 2011). We selected the whole Iberian Peninsula as background for all models to avoid problems derived from using different study areas when evaluating model performance with AUC (Jiménez‐Valverde et al. 2008). In addition, the whole Iberian Peninsula is the most accessible area to the genetic units considered in the study (Barve et al. 2011) since the Pyrenees form an important natural barrier with the rest of Europe. We managed to reduce the extent of spatial autocorrelation in model residuals (as in Marcer et al. 2012), but at a high cost of data reduction (e.g., up to 80% of reduction in the number of accessions for the species and ending with less than 25 accessions in some genetic groups). Given that the objective is the relative measures of accuracy between genetic units rather than absolute accuracy, we finally ran our models without spatial or environmental data filtering and accept that absolute measures of model accuracy (AUC) may be somehow inflated (Veloz 2009). The reported AUC is the average test AUC given by MaxEnt and resulting from a 10‐fold cross‐validation. Lastly, we ran all models again with the whole set of accessions to get the final models.

Based on MaxEnt predictive maps, we estimated niche breadth for each genetic unit for present and future predicted environmental conditions as in Warren et al. (2008) and Banta et al. (2012). Niche breadth, which ranges between 0 (the narrowest niche breadth) and 1 (the maximum possible niche breadth), gives an indication of the species’ tolerance to varying environments, which in turn determines the species’ potential distribution range (Banta et al. 2012 and references therein). We also obtained the environmental suitability score, which varies between 0 and 1, for each *A. thaliana* accession; that is, a measure of the favorability of accessions to environmental variables in their grid cells. The variables that mattered most for the distribution of each genetic unit were identified by their relative contributions, given as percentages, to the fit of the models. These were generated by the MaxEnt's jackknife procedure, which compares the training gain of each variable in isolation to the training gain of the model with all variables.

We also identified the environmental variables underlying the divergence in potential distribution ranges between genetic units of *A. thaliana*. To this end, we generated kernel density plots (as in Theodoridis et al. 2013) to visualize the distribution of predicted occurrence cells for each environmental variable and genetic unit. We used all occurrence cells after tallying model probabilities with a threshold suitability score of 0.85. After trying several incremental thresholds, it was at this threshold when we started seeing a separation between variables for each genetic unit. Differences between genetic units were estimated by comparing magnitudes of Cohen's *d*, which measures the standardized difference between two means (Cohen 1988), applied to kernel density plots for each genetic unit and environmental variable. As we are dealing with the whole population of measures, all grid cell values, there is no need to perform statistical tests to evaluate them. For the sake of clarity, we only interpreted those environmental variables that clearly differentiated OVR categories, nuclear genetic clusters, or chlorotype genetic groups from each other (i.e., low overlap between kernel density plots).

## Results

### Current potential distribution ranges

**Figure 1:**
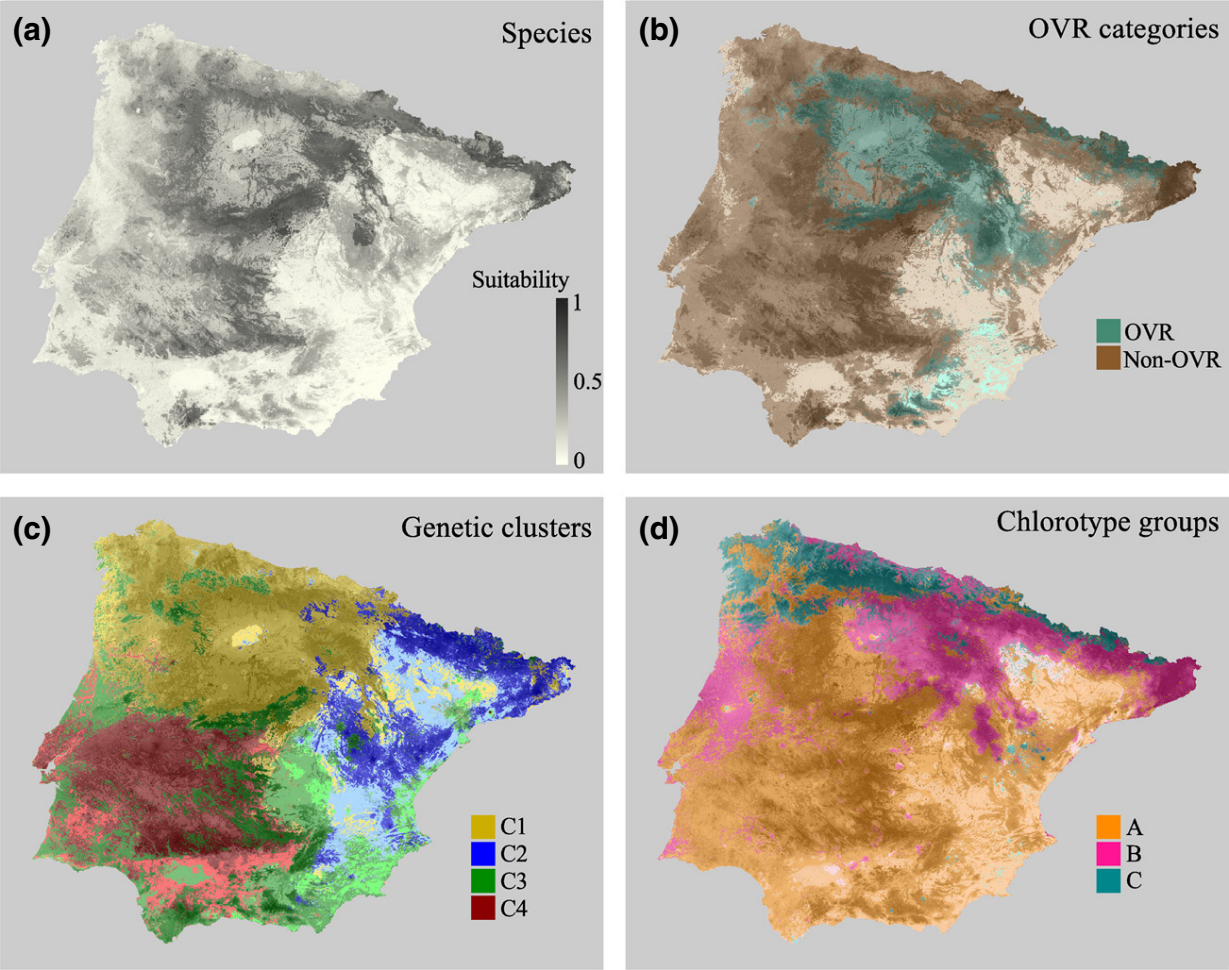
Current potential distribution range of *Arabidopsis thaliana* in the Iberian Peninsula for (a) the whole‐species level, (b) phenotypic categories (OVR and non‐OVR), (c) nuclear genetic clusters (C1, C2, C3, and C4), and (d) chlorotype genetic groups (A, B, and C). Darker and lighter intensities for each color indicate higher and lower suitability, respectively.

Potential distribution ranges were estimated for *A. thaliana* at the species level and for the different genetic units based on flowering phenotypes (OVR and non‐OVR), nuclear genetic clusters and chlorotype groups. AUC test values (Table 1) for current potential distribution ranges (Fig. 1) were generally higher for genetic units (average AUC tests among genetic units = 0.83) than at the species level (AUC test = 0.77), indicating a better accuracy of the model when MaxEnt was applied to genetic units (Table 1). Only AUC test values for chlorotype A (AUC test = 0.68) and non‐OVR category (AUC test = 0.76) were lower than the AUC test value at the species level.

**Table 1:** Species distribution models performance at the whole‐species and genetic‐unit levels given by AUC test values (±SD). Percentage changes between whole‐species’ and genetic units’ AUC test values (ΔAUC) are also given. Negative and positive ΔAUC indicates decreases and increases in AUC test values with respect to the species’ AUC test value, respectively

Unit	Level	AUC test value	ΔAUC (%)
Species	–	0.766 ± 0.039	–
Phenotypic category	Non‐OVR	0.764 ± 0.030	−0.26
Phenotypic category	OVR	0.874 ± 0.038	14.10
Genetic cluster	C1	0.854 ± 0.044	11.49
Genetic cluster	C2	0.917 ± 0.041	19.71
Genetic cluster	C3	0.834 ± 0.081	8.88
Genetic cluster	C4	0.869 ± 0.097	13.45
Chlorotype group	A	0.682 ± 0.060	−10.97
Chlorotype group	B	0.805 ± 0.097	5.09
Chlorotype group	C	0.866 ± 0.133	13.05

### Environmental variable contribution

The potential distribution range of the species (Fig. 1a) was mainly explained by pH and the percentage of agricultural land (Table 2). The species was more likely to occur in acidic areas of the Iberian Peninsula (*N *=* *279; mean pH ± SE = 5.66 ± 0.05) and habitats with lower percentages of agricultural land (*N *=* *279; mean percentage of agricultural land ± SE = 33.94 ± 2.14%).

**Table 2:** Environmental variable percent contribution to the fit of the models. Climatic variables: BIO1, annual mean temperature; BIO2, mean diurnal temperature range; BIO4, temperature seasonality; BIO12, annual precipitation; BIO15, precipitation seasonality. The largest contributions summing more than 50% are given in bold face

	Species	Phenotypic categories		Genetic clusters				Chlorotype groups		
Variable	–	Non‐OVR	OVR	C1	C2	C3	C4	A	B	C
BIO1	18.83	**15.67**	**66.32**	**38.62**	7.98	4.27	7.11	14.87	4.28	**69.23**
BIO2	0.81	0.53	0.08	2.23	1.16	0.54	0.09	2.65	0.75	0.02
BIO4	1.99	2.95	8.17	1.31	0.84	17.50	**24.24**	9.29	4.35	15.62
BIO12	5.55	12.26	0.37	2.59	4.55	0.30	1.25	10.05	18.55	0.03
BIO15	2.09	6.60	1.24	1.95	**43.62**	**20.03**	**43.67**	5.85	**41.19**	6.48
pH	**47.39**	**42.06**	10.67	**40.69**	13.91	10.38	16.45	**32.90**	**20.03**	1.10
% Agriculture	**20.35**	13.53	12.69	3.87	**27.23**	**45.58**	6.40	**18.37**	9.29	7.16
% Urban	2.99	6.40	0.46	8.74	0.71	1.39	0.78	6.03	1.56	0.35

As far as OVR categories are concerned (Fig. 1b), the potential distribution range of OVR accessions was accounted for by annual mean temperature (Table 2). OVR accessions were more likely to occur in colder environments (*N *=* *82; mean BIO1 ± SE = 10.25 ± 0.22°C; Table S2). The potential distribution range of non‐OVR accessions was determined by pH and to a lesser extent by annual mean temperature (Table 2). Non‐OVR accessions were more likely to occur in more acidic soils (*N *=* *197; mean pH ± SE = 5.56 ± 0.05; Table S2) and warmer environments (*N *=* *197; mean BIO1 ± SE = 13.02 ± 0.17°C; Table S2).

The potential distribution ranges of nuclear genetic clusters (Fig. 1c) were also explained by different environmental variables. Cluster C1 was accounted for by pH and annual mean temperature (Table 2), with more acidic soils (*N *=* *116; mean pH ± SE = 5.39 ± 0.06; Table S2) and colder annual mean temperatures (*N *=* *116; mean BIO1 ± SE = 11.72 ± 0.14°C; Table S2) increasing the occurrence probability of C1 accessions. Cluster C2 was explained by precipitation seasonality and percentage of agricultural land (Table 2), with C2 accessions increasing their occurrence probability in areas with lower precipitation seasonality (*N *=* *36; mean BIO15 ± SE = 26.16 ± 1.14; Table S2) and lower percentage of agricultural land (*N *=* *36; mean percentage of agricultural land ± SE = 14.28 ± 3.84%; Table S2). Cluster C3 was also accounted for by the percentage of agricultural land and precipitation seasonality (Table 2), with C3 accessions increasing their occurrence probability in areas with lower agricultural land (*N *=* *31; mean percentage of agricultural land ± SE = 13.60 ± 4.55%; Table S2) and higher precipitation seasonality (*N *=* *31; mean BIO15 ± SE = 47.40 ± 2.04; Table S2). The potential distribution range of cluster C4 was mainly explained by precipitation seasonality and temperature seasonality (Table 2), with C4 accessions occurring with higher probability in areas with higher seasonality in precipitation (*N *=* *29; mean BIO15 ± SE = 57.40 ± 1.45; Table S2) and higher seasonality in temperature (*N *=* *29; mean BIO4 ± SE = 6.28 ± 0.68; Table S2).

Finally, the potential distribution ranges of the three chlorotype groups (Fig. 1d) were explained as follows. Group A was accounted for by pH and the percentage of agricultural land (Table 2), with A accessions more likely to occur in less acidic areas (*N *=* *102; mean pH ± SE = 5.63 ± 0.09; Table S2) and higher agricultural land (*N *=* *102; mean percentage of agricultural land ± SE = 39.10 ± 3.61%; Table S2). Group B was mainly explained by precipitation seasonality and pH (Table 2), with B accessions occurring with higher probability in areas with lower precipitation seasonality (*N *=* *63; mean BIO15 ± SE = 32.22 ± 1.49; Table S2) and less acidic soils (*N *=* *63; mean pH ± SE = 5.73 ± 0.10; Table S2). Group C was accounted for by annual mean temperature (Table 2), with C accessions occurring mostly in colder environments (*N *=* *16; mean BIO1 ± SE = 9.81 ± 0.62°C; Table S2).

### Environmental variable separation of genetic units

**Figure 2:**
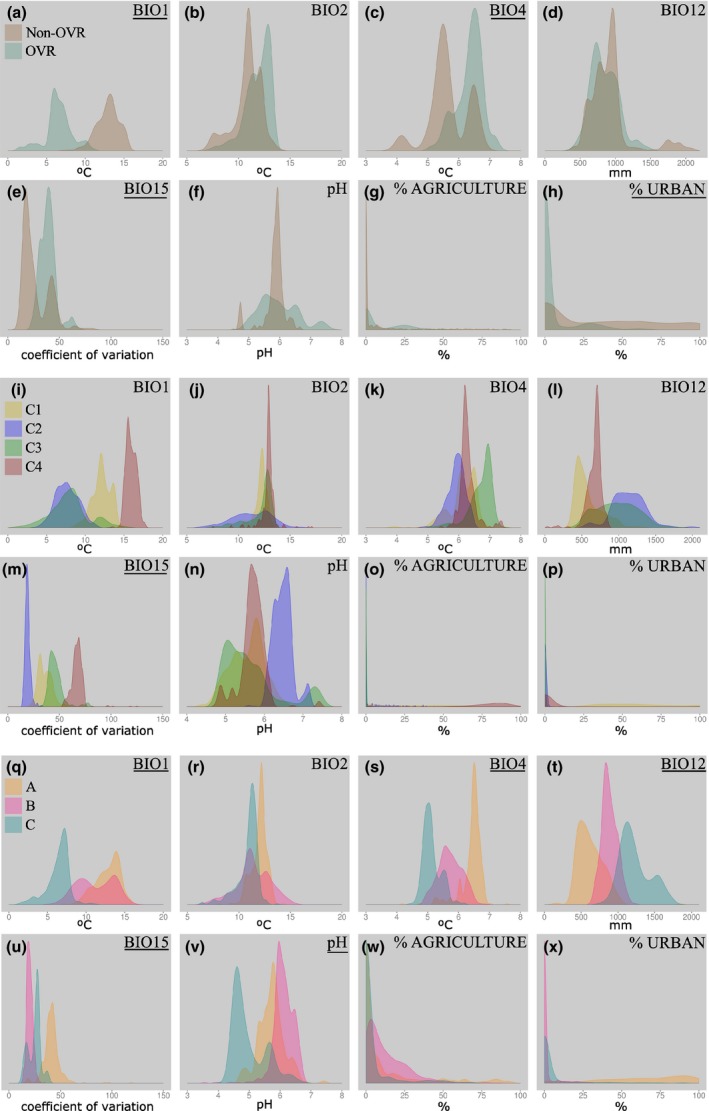
Kernel density plots for the five climatic variables, pH and two land cover variables for phenotypic categories (OVR and non‐OVR), nuclear genetic clusters (C1, C2, C3, and C4), and chlorotype genetic groups (A, B, and C). Underlined variable names indicate that OVR categories, nuclear genetic clusters or chlorotype genetic groups were distinguishable from each other, based on Cohen's *d* (Table S1), for that particular environmental variable. See codes for climatic variables in Table 2.

Based on Cohen's *d* values applied to the distribution of predicted occurrence cells of each environmental variable for each genetic unit (Fig. 2, Table S2), the environmental variables differentiating the potential distribution ranges between OVR categories, nuclear genetic clusters, or chlorotype genetic groups from each other (i.e., low overlap between kernel density plots) were the following. First, OVR and non‐OVR categories differed in annual mean temperature, temperature seasonality, and precipitation seasonality (Fig. 2a,c,e and h). Second, precipitation seasonality was the only environmental variable that clearly differentiated the four nuclear genetic clusters from each other (Fig. 2m). Third, chlorotype groups differed from one another in annual mean temperature, temperature seasonality, annual precipitation, precipitation seasonality, and pH (Fig. 2q,s,t,u, and v).

### Niche breadth and suitability scores with GCC

**Figure 3:**
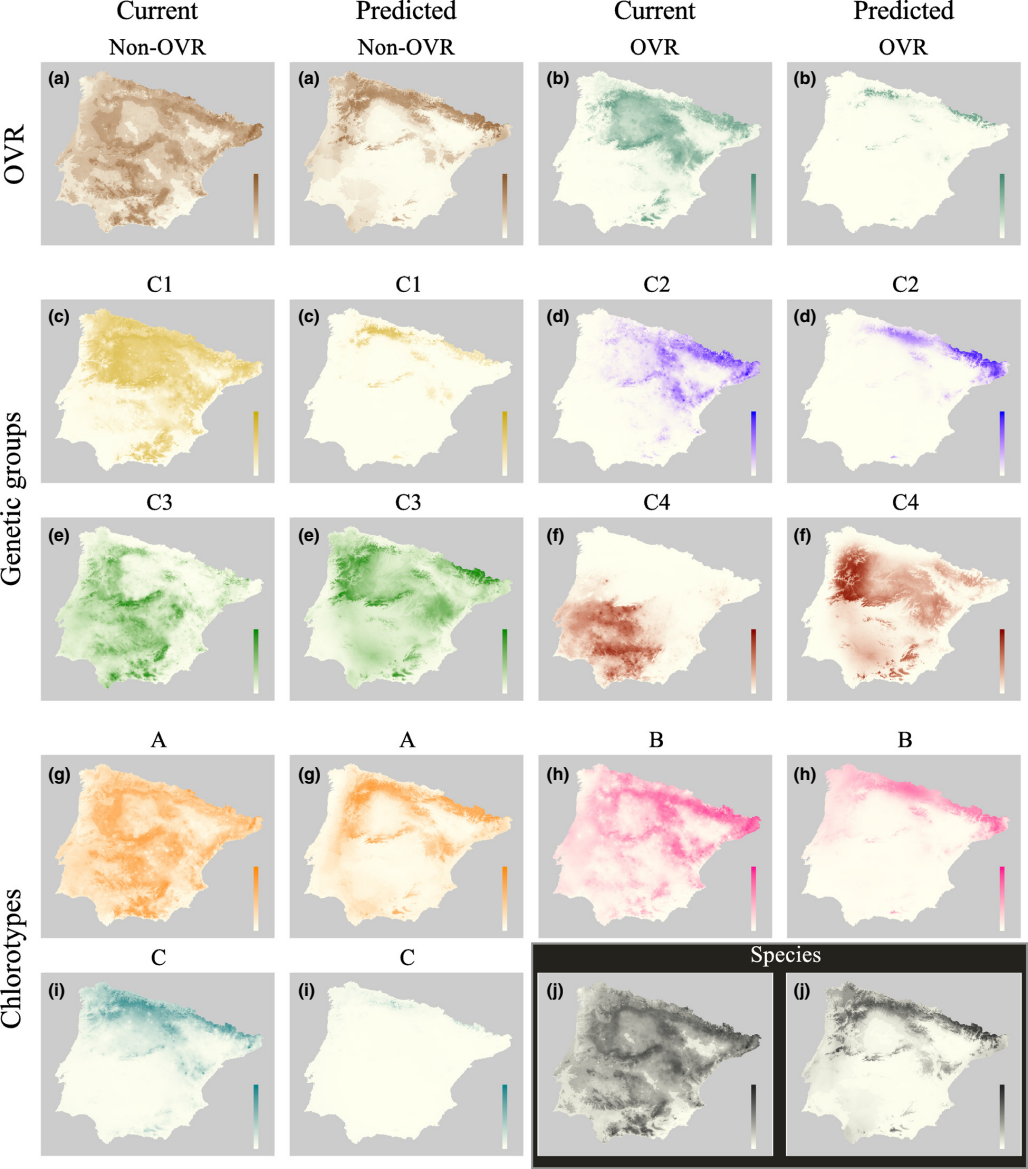
Current and future potential distribution ranges for the species as a whole, phenotypic categories (OVR and non‐OVR), nuclear genetic clusters (C1, C2, C3, and C4), and chlorotype genetic groups (A, B, and C). Darker and lighter intensities for each color indicate higher and lower suitability, respectively.

Here, we use niche breadth (as in Banta et al. 2012) as a measure of present and future potential spatial distribution range expansions or contractions. It should be interpreted not as a static property of a species or genetic unit, which stems from its Grinnellian fundamental niche (Soberón 2007), but as a way to quantify the broadness of environmental conditions tolerated by the species given the set of predictors used (a subset of the fundamental niche). The comparison of potential distribution ranges between current and future climatic conditions using the 2070 RCP8.5 greenhouse gas concentration scenario indicated that *A. thaliana* might reduce its niche breadth in the Iberian Peninsula up to 57% (Table 3, Fig. 3j). Future projections also showed that suitability scores of the 279 study accessions might decrease up to 73.2% (Table 3) with the future climatic condition predicted by the 2070 RCP8.5 scenario.

**Table 3:** Suitability scores and niche breadths at the whole‐species and genetic‐unit levels. Mean suitability scores (SC ± SD), total niche breadths (NB), and percentage changes in SC (ΔSC) and NB (ΔNB) between current and future scenarios and given

Unit	Subunit	Current SC	Future SC	ΔSC (%)	Current NB	Future NB	ΔNB (%)
Species	–	0.518 ± 0.162	0.139 ± 0.210	−73.16	0.808	0.347	−57.05
Phenotypic category	Non‐OVR	0.528 ± 0.171	0.216 ± 0.224	−59.09	0.807	0.406	−49.75
Phenotypic category	OVR	0.529 ± 0.178	0.087 ± 0.156	−83.55	0.402	0.065	−83.83
Genetic cluster	C1	0.548 ± 0.135	0.048 ± 0.111	−91.24	0.601	0.109	−81.86
Genetic cluster	C2	0.570 ± 0.281	0.411 ± 0.309	−27.89	0.283	0.105	−62.90
Genetic cluster	C3	0.551 ± 0.280	0.427 ± 0.249	−22.50	0.594	0.655	10.27
Genetic cluster	C4	0.545 ± 0.210	0.147 ± 0.093	−73.03	0.317	0.423	33.44
Chlorotype group	A	0.539 ± 0.165	0.212 ± 0.205	−60.67	0.810	0.495	−38.89
Chlorotype group	B	0.536 ± 0.225	0.275 ± 0.236	−48.69	0.607	0.279	−54.04
Chlorotype group	C	0.566 ± 0.269	0.052 ± 0.064	−90.81	0.362	0.077	−78.73

Comparisons for each OVR category indicated that niche breadths and suitability scores for non‐OVR accessions decreased 49.8% and 59.1%, respectively, with the GCC scenario (Table 3, Fig. 3a). In the case of OVR accessions, they exhibited a more pronounced reduction in both niche breadth (83.8%) and suitability scores (83.6%; Table 3, Fig. 3b). For nuclear genetic clusters, niche breadths behaved differently when comparing current and future climatic conditions predicted by the 2070 RCP8.5 scenario (Table 3, Fig. 3c–f). Clusters C1 and C2 showed the most marked reduction in niche breadth (81.9% and 62.9%, respectively), whereas clusters C3 and C4 exhibited increases in their niche breadths (10.3 and 33.4% for C3 and C4, respectively) with the predicted climatic conditions. Percentage changes in suitability scores were all negative for all four genetic clusters (Table 3), with very high reductions for clusters C1 (91.2%) and C4 (73.0%) and moderate for C2 (27.9%) and C3 (22.5%). Finally, all chlorotype groups exhibited moderate‐to‐high shrinkages in niche breadth (38.9, 54.0, and 78.7% for groups A, B, and C, respectively) as well as moderate‐to‐high reductions in suitability scores (60.7, 48.7, and 90.8% for groups A, B, and C, respectively) with the 2070 RCP8.5 scenario (Table 3, Fig. 3g–i).

Global climate change effects, as given by suitability scores and niche breadth shifts, have to be interpreted in the context of the five climatic variables used in these models, as future GCC scenarios lack land use and soil data. In this case, annual mean temperature, precipitation seasonality, and temperature seasonality were the climatic variables with the most important contributions to the fit of the models (Table S3).

## Discussion

We have shown that decomposing a species into intraspecific genetic units increases our understanding of potential range fluctuations under a GCC scenario in line with other studies (D'Amen et al. 2013; Oney et al. 2013; Gotelli and Stanton‐Geddes 2015). In general, SDM performed more accurately in estimating potential distribution range when using data from genetic units rather than from the species as a whole (range of accuracy increases = 9–20%). Lower accuracies were obtained for genetic units showing wide distribution ranges like the whole‐species approach (i.e., non‐OVR category and chlorotype genetic group A), adding to the findings that more common species tend to have lower values of predictive accuracy (Allouche et al. 2006; Acevedo et al. 2012). Hence, our results reinforce the view that wide‐range species can be highly heterogeneous entities as a result of environmentally driven demographic and/or adaptive processes. Intraspecific genetic units are more environmentally tuned compact units, and hence, they can better help discern environmental drivers that may affect differently the genetic units that form a species. Consequently, working with intraspecific genetic units may provide a better understanding of the effects of GCC on the potential future distribution of the species as a whole.

Our approach allowed the disentanglement of the environmental variables correlating with, and possibly accounting for, the potential distribution range at the whole‐species and genetic‐unit levels. The potential distribution range at the whole‐species level was mainly explained by variation in pH, percentage of agricultural land, and annual mean temperature. On the other hand, genetic units with better distribution model accuracies exhibited different combinations of environmental variables underpinning their potential distribution ranges. Clearly, pH, agricultural land, and annual mean temperature were involved in fitting the models, although precipitation seasonality also had a relevant role. It must be noted that precipitation seasonality was the only variable that clearly differentiated all genetic units based on the three criteria as shown by kernel density plots. Interestingly, the ecological weight that precipitation seasonality may have in *A. thaliana*'s distribution is also supported by a recent study on adaptive variation and its environmental drivers indicating that precipitation seasonality was a good predictor for individual fitness components (Manzano‐Piedras et al. 2014). Thus, SDM applied to intraspecific genetic units can also be regarded as a methodological contribution for the detection of environmental variables accounting for geographical genetic structure.

Dealing with intraspecific genetic units definitely allows a deeper understanding of GCC‐driven changes in genetic diversity (Yannic et al. 2014). For example, potential distribution ranges of the two categories of accessions differing in their obligate vernalization requirement for flowering (i.e., OVR and non‐OVR) conferred more realism to the approach as they occupied different geographical ranges determined by different environmental variables. OVR accessions occur in colder environments, whereas non‐OVR ones are more ubiquitous across the Iberian Peninsula. Given that the GCC scenario chosen in this study predicts warmer environments in the near future, OVR accessions might have more limited distributions in the Iberian Peninsula and they should adapt to the new environmental conditions to persist over time. A common garden experiment with the same set of 279 *A. thaliana* accessions recently showed that accessions from cold environments were able to complete the life cycle in a warmer environment but with lower fitness performance (Manzano‐Piedras et al. 2014). Thus, it is reasonable to predict a progressive disappearance of the obligate vernalization requirement for flowering in *A. thaliana* in a warmer and drier Iberian Peninsula.

We had previously shown that Iberian OVR accessions only occur above 800 m in environments with annual mean temperatures below 5°C and that accessions from higher altitudes exhibit a late‐flowering behavior (Méndez‐Vigo et al. 2011). Hence, GCC under the 2070 RCP8.5 scenario and the dramatic shrinkage predicted for the potential distribution range of OVR accessions might erase gene polymorphisms typically associated to late flowering in *A. thaliana* in Iberian cold environments, which likely confer local adaptation to such environments (Méndez‐Vigo et al. 2011; Banta et al. 2012). This conclusion is also supported by a recent study applying climate envelope models to early‐ and late‐flowering *A. thaliana* genotypes in Eurasia showing that flowering time and potential distribution range were negatively correlated, which in turn constrained the distribution of various loci associated to late‐flowering time (Banta et al. 2012). Thus, SDM applied to ecologically and evolutionarily important traits can be regarded as a tool that may help identify functional genetic diversity whose adaptive potential is threatened by GCC.

Nuclear genetic clusters and chloroplast genetic groups also provide new insight into the factors underlying observed shifts in *A. thaliana*'s potential distribution range. Genetic clusters are based on neutral genomic SNP markers, meaning that demographic history is expected to be primarily responsible for such genetic structure. However, local adaptation does exist in *A. thaliana* (McKay et al. 2003; Kover et al. 2009; Fournier‐Level et al. 2011; Méndez‐Vigo et al. 2011, 2013; Ågren and Schemske 2012; Kronholm et al. 2012; Brachi et al. 2013; Manzano‐Piedras et al. 2014; Wilczek et al. 2014; Hamilton et al. 2015), which allows for correlative models, such as SDM, to be applied to the genetic clusters derived from neutral markers. Chloroplast genetic groups give us an indication about the geographic distribution of maternally inherited genetic variation. Almost all nuclear genetic clusters and chloroplast genetic groups exhibited important shrinkages in their potential distribution ranges with the 2070 RCP8.5 scenario. Only genetic clusters C3 and C4 increased their potential distribution ranges with pronounced northward range shifts. Thus, *A. thaliana* from different genetic clusters is expected to encounter environmental conditions that may determine contractions or spreads of its potential distribution range with GCC, which can be interpreted as the product of different combinations of environmental variable shifts. SDM applied to nuclear genetic clusters and chloroplast genetic groups allow for the identification of those units that may be more threatened but also those that may buffer environmental change under GCC, resulting in shifts of the relative proportions of the species geographical extent occupied by each genetic unit. It is worth noting that genetic units behave like species with smaller ranges, which are more susceptible to GCC (Pauls et al. 2013). Hence, SDM applied to intraspecific genetic units may be more realistic in forecasting the effects of GCC on the genetic composition of a species in future scenarios by treating species as an assemblage of smaller‐range genetic units better tuned to their specific environments.

Our results stress the need to combine complementary sources of intraspecific genetic variation to obtain a comprehensive picture of how biogeographical patterns and genetic diversity can be affected by GCC‐driven range fluctuations. Here, we have only quantified the predicted change/loss of genetic diversity with GCC under the 2070 RCP8.5 scenario. In order to quantify change/loss of genetic diversity with GCC, comparisons with multiple GCC scenarios and different approaches to estimate it would be required. Overall, in our case and for illustrative purposes, *A. thaliana*'s genetic diversity is expected to shift toward non‐OVR phenotypes with genetic backgrounds mostly represented by nuclear genetic clusters C3 and C4 and chlorotype genetic group A.

Species distribution models based on genetic units may provide a powerful tool for conservation managers to improve the decision‐making process when facing threats to regional biodiversity. Managers would acquire a better understanding of the scenarios of loss of genetic diversity by identifying those populations and region‐specific genetic variants at higher risk of extinction but also those that may thrive with GCC, which would greatly help in the conservation decision‐making process (Maxted et al. 2008; Thomassen et al. 2010b; Rivers et al. 2014). In particular, our suggestion is to focus on two sources of intraspecific genetic variation with particularly high conservation value. First, phenotypic categories for key life‐cycle traits are particularly interesting because they are generally under strong selective pressure (e.g., flowering time in *A. thaliana*; Méndez‐Vigo et al. 2013). Second, genetic clusters based on co‐dominant nuclear markers are also important because they mostly provide a clear picture on the recent species’ demographic history across the study region.

Finally, we want to outline some caveats that need to be heeded in order to develop the right tools to reduce model uncertainty and make better predictions. First, our models identified soil and land use variables that contributed significantly to their fit. However, GCC scenarios only take climatic variables into account, which may bias predictions. Impacts of GCC on soil properties and land use (Singh et al. 2011; Brevik 2012; EEA 2012) should be considered in this sort of studies. Second, SDM were designed to be used with binary data but genetic data tend to be continuous (e.g., proportion of individuals requiring OVR for each genotype, percentage of cluster membership). In this study, we have categorized our data but ignore whether we have lost resolution and statistical power when converting continuous to binary data. Similar models based on continuous data have to be developed and outcomes compared to those of binary SDM. Third, in the particular case of *A. thaliana*, currently available genome‐wide data (Weigel 2012) will allow the analyses of intraspecific genetic units based on gene network variation for various evolutionarily important phenotypic traits (e.g., flowering time, seed dormancy). This novel approach would provide the means to assess the extent to which key functional genetic variation may be threatened by GCC scenarios (Banta et al. 2012). Finally, the most difficult challenge is to combine demographic and genetic models with SDM to better predict the spatiotemporal response of intraspecific genetic levels to GCC (Hoffmann and Sgrò 2011; Brown and Knowles 2012; Fordham et al. 2014; Gavin et al. 2014; Merow et al. 2014). It is important to couple migration patterns with stochastic environmental changes as well as with the rapid evolutionary changes in traits that may determine population performance with GCC. Although such multidisciplinary methods are currently under an intensive conceptual and technical development, we urgently need new models based on high‐density occurrence datasets and various sources of genetic/genomic variation characterizing demographic patterns and the adaptive history of study species.

## Data accessibility

Data available from the Dryad Digital Repository: http://dx.doi.org/10.5061/dryad.vv804.

## Conflict of Interest

None declared.

## Supporting information


**Figure S1.** Spatial distribution of Iberian *Arabidopsis thaliana* accessions based on genetic units: OVR categories (*N *=* *279), genetic clusters (*N *=* *212) and chlorotype groups (*N *=* *181).
**Figure S2.** Genetic structure of Iberian *Arabidopsis thaliana* accessions estimated with STRUCTURE and nuclear SNPs. Accessions are depicted as horizontal bars divided in segments representing the estimated membership proportions of genetic clusters (*K*) fitted in the model. Yellow, blue, green and red depict genetic clusters C1, C2, C3 and C4, respectively. Accessions are arranged according to estimated cluster memberships proportions for *K *=* *4.
**Figure S3.** Chlorotype network of *Arabidopsis thaliana* accessions estimated with NETWORK. Chlorotype groups (A, B, and C) include closely related chlorotypes for the sake of simplicity. Each branch corresponds to one mutational step between chlorotypes. Non‐observed mutational steps between chlorotypes are indicated by perpendicular dashes. Circle size is proportional to the number of accessions within chlorotypes.
**Table S1.** Cohen's *d* and differences between OVR categories, nuclear genetic clusters and chlorotype genetic groups for each environmental variable.
**Table S2.** Mean (±SE) altitude and mean (±SE) values for genetic units and environmental variables included in SDM.
**Table S3.** Climatic variable percent contribution to the fit of the models.
