# Supplementary material for: Tackling intraspecific genetic structure in distribution models better reflects species geographical range
Source: Ecol Evol. 2016 Feb 26;6(7):2084–97. doi: 10.1002/ece3.2010 (PMC4768750; doi:10.1002/ece3.2010)
Supplement: Supplementary file 1 — Figure S1. Spatial distribution of Iberian Arabidopsis thaliana accessions based on genetic units: OVR categories (N = 279), genetic clusters (N = 212) and chlorotype groups (N = 181). Figure S2. Genetic structure of Iberian Arabidopsis thaliana accessions estimated with STRUCTURE and nuclear SNPs. Accessions are depicted as horizontal bars divided in segments representing the estimated membership proportions of genetic clusters (K) fitted in the model. Yellow, blue, green and red depict genetic clusters C1, C2, C3 and C4, respectively. Accessions are arranged according to estimated cluster memberships proportions for K = 4. Figure S3. Chlorotype network of Arabidopsis thaliana accessions estimated with NETWORK. Chlorotype groups (A, B, and C) include closely related chlorotypes for the sake of simplicity. Each branch corresponds to one mutational step between chlorotypes. Non‐observed mutational steps between chlorotypes are indicated by perpendicular dashes. Circle size is proportional to the number of accessions within chlorotypes. Table S1. Cohen's d and differences between OVR categories, nuclear genetic clusters and chlorotype genetic groups for each environmental variable. Table S2. Mean (±SE) altitude and mean (±SE) values for genetic units and environmental variables included in SDM. Table S3. Climatic variable percent contribution to the fit of the models. [file ECE3-6-2084-s001.docx]

**Figure S1.** Spatial distribution of Iberian *Arabidopsis thaliana* accessions based on genetic units: OVR categories (*N* = 279), genetic clusters (*N* = 212) and chlorotype groups (*N* = 181).


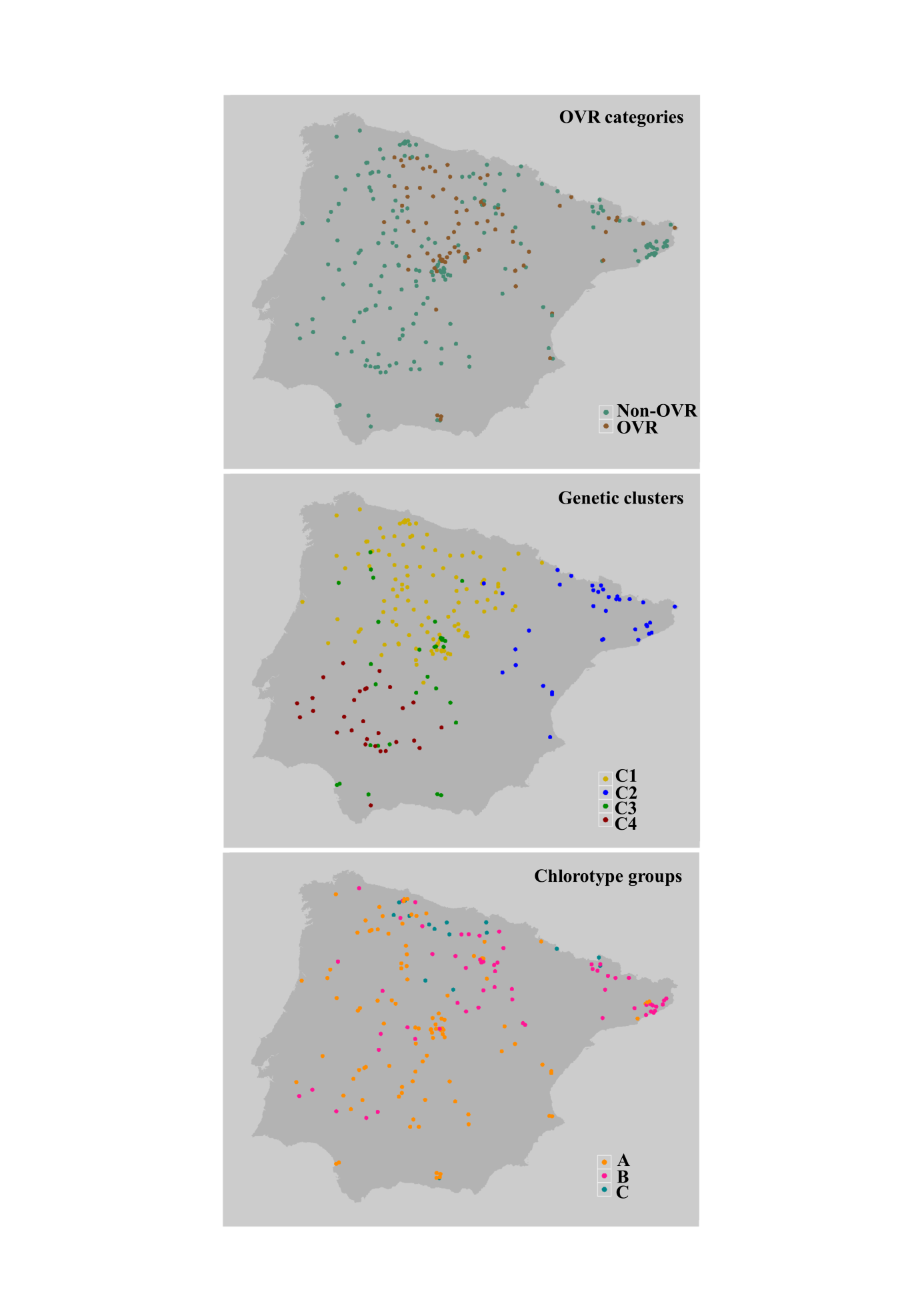


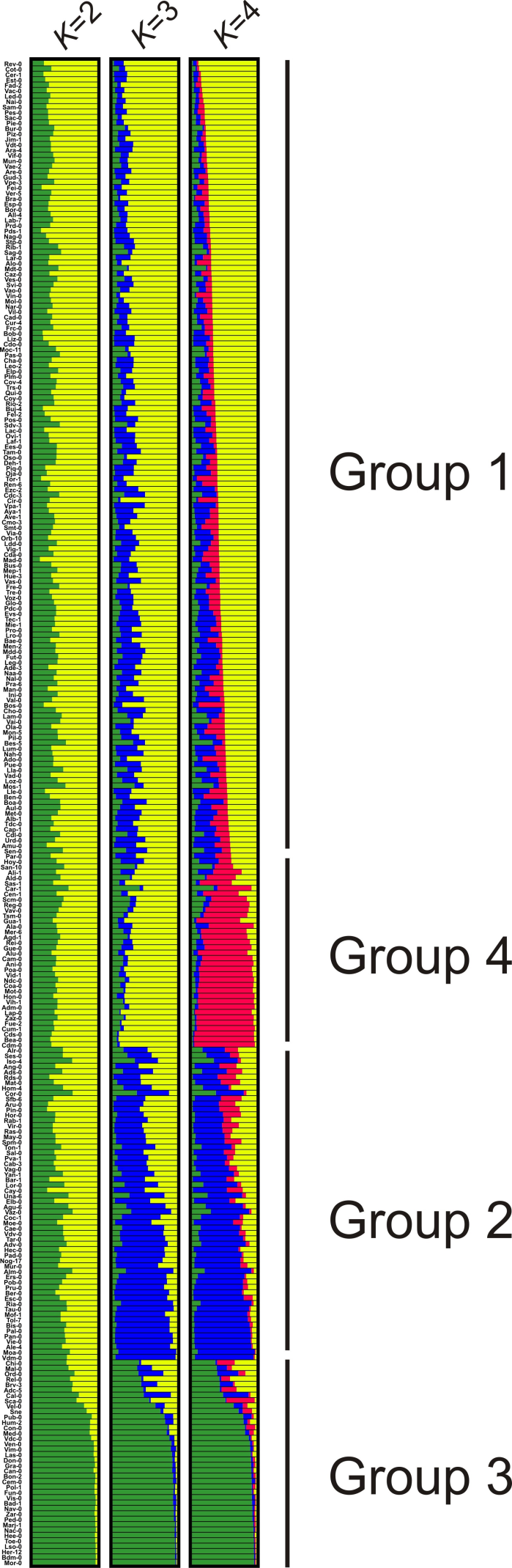


**Figure S2.** Genetic structure of Iberian *Arabidopsis thaliana* accessions estimated with STRUCTURE and nuclear SNPs. Accession are depicted as horizontal bars divided in segments representing the estimated membership proportions of genetic clusters (*K*) fitted in the model. Yellow, blue, green and red depict genetic clusters C1, C2, C3 and C4, respectively. Accessions are arranged according to estimated cluster memberships proportions for *K*=4.

**Figure S3.** Chlorotype network of *Arabidopsis thaliana* accessions estimated with NETWORK. Chlorotype groups (A, B, and C) include closely related chlorotypes for the sake of simplicity. Each branch corresponds to one mutational step between chlorotypes. Non-observed mutational steps between chlorotypes are indicated by perpendicular dashes. Circle size is proportional to the number of accessions within chlorotypes.


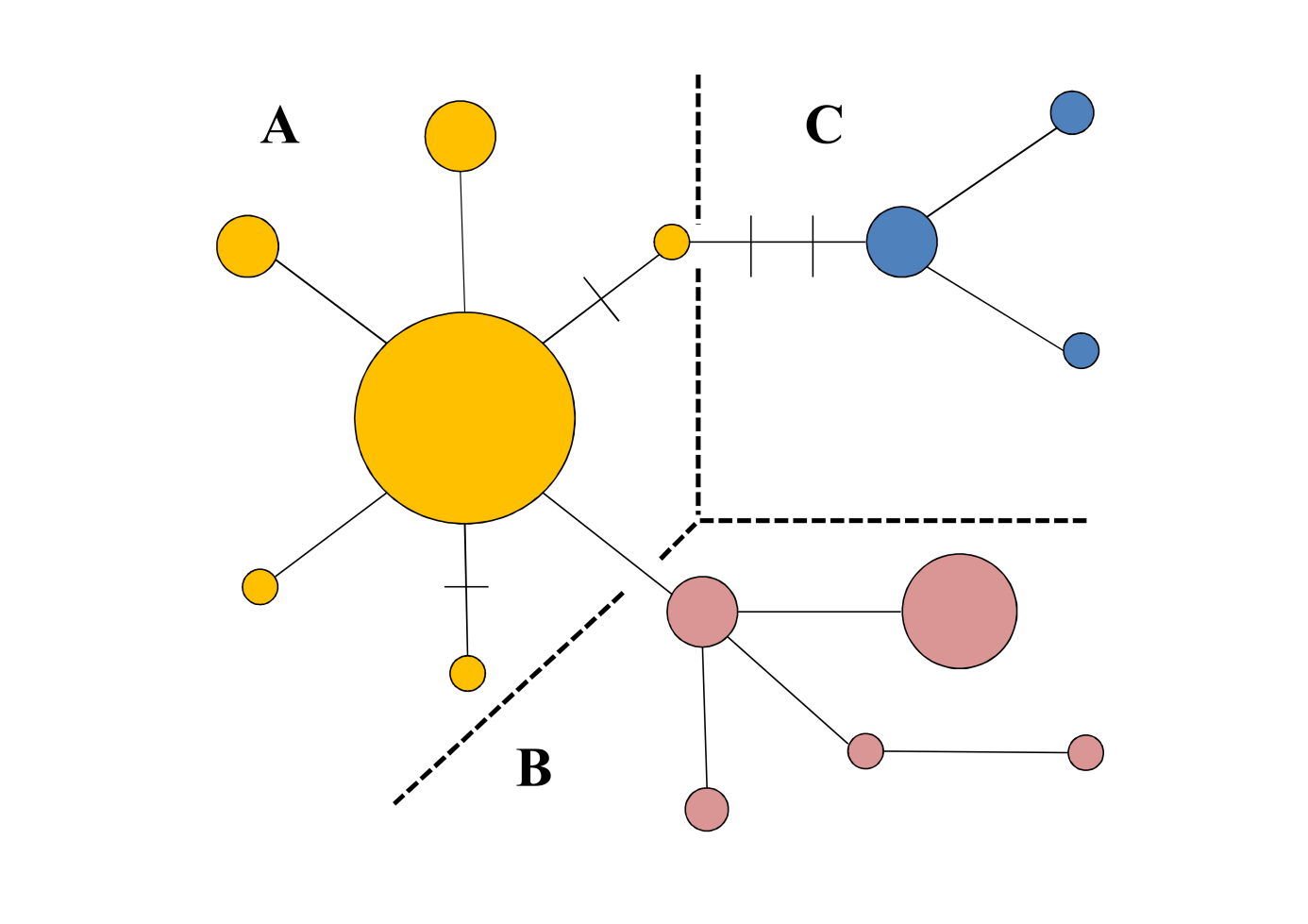


**Table S1.** Cohen’s *d* and and subsequent differences between OVR categories, nuclear genetic clusters and chlorotype genetic groups for each environmental variable.

| Genetic Unit | Variable | Level L1 | Level L2 | Cohen’s *d* | L1 *vs*. L2 |
| --- | --- | --- | --- | --- | --- |
| OVR category | BIO1 | OVR | Non-OVR | -4.120 ± 0.331 | 1 <<< 2 |
| OVR category | BIO2 | OVR | Non-OVR | 0.790 ± 0.257 | 1 >> 2 |
| OVR category | BIO4 | OVR | Non-OVR | 1.206 ± 0.262 | 1 >>> 2 |
| OVR category | BIO12 | OVR | Non-OVR | -0.245 ± 0.253 | 1 < 2 |
| OVR category | BIO15 | OVR | Non-OVR | 1.076 ± 0.261 | 1 >>> 2 |
| OVR category | pH | OVR | Non-OVR | 0.282 ± 0.253 | 1 > 2 |
| OVR category | % Agriculture | OVR | Non-OVR | 0.029 ± 0.252 | 1 ~ 2 |
| OVR category | % Urban | OVR | Non-OVR | -0.981 ± 0.259 | 1 <<< 2 |
| Genetic cluster | BIO1 | C1 | C2 | 3.424 ± 0.282 | 1 >>> 2 |
| Genetic cluster | BIO1 | C1 | C3 | 2.130 ± 0.237 | 1 >>> 2 |
| Genetic cluster | BIO1 | C1 | C4 | -3.362 ± 0.302 | 1 <<< 2 |
| Genetic cluster | BIO1 | C2 | C3 | -0.162 ± 0.217 | 1 ~ 2 |
| Genetic cluster | BIO1 | C2 | C4 | -6.490 ± 0.388 | 1 <<< 2 |
| Genetic cluster | BIO1 | C3 | C4 | -3.725 ± 0.296 | 1 <<< 2 |
| Genetic cluster | BIO2 | C1 | C2 | 0.778 ± 0.230 | 1 >> 2 |
| Genetic cluster | BIO2 | C1 | C3 | 0.139 ± 0.212 | 1 ~ 2 |
| Genetic cluster | BIO2 | C1 | C4 | -0.627 ± 0.249 | 1 << 2 |
| Genetic cluster | BIO2 | C2 | C3 | -0.585 ± 0.219 | 1 << 2 |
| Genetic cluster | BIO2 | C2 | C4 | -1.013 ± 0.257 | 1 <<< 2 |
| Genetic cluster | BIO2 | C3 | C4 | -0.524 ± 0.242 | 1 << 2 |
| Genetic cluster | BIO4 | C1 | C2 | 0.638 ± 0.228 | 1 >> 2 |
| Genetic cluster | BIO4 | C1 | C3 | -1.216 ± 0.221 | 1 <<< 2 |
| Genetic cluster | BIO4 | C1 | C4 | -0.110 ± 0.247 | 1 ~ 2 |
| Genetic cluster | BIO4 | C2 | C3 | -2.183 ± 0.243 | 1 <<< 2 |
| Genetic cluster | BIO4 | C2 | C4 | -1.144 ± 0.259 | 1 <<< 2 |
| Genetic cluster | BIO4 | C3 | C4 | 1.285 ± 0.249 | 1 >>> 2 |
| Genetic cluster | BIO12 | C1 | C2 | -2.693 ± 0.265 | 1 <<< 2 |
| Genetic cluster | BIO12 | C1 | C3 | -1.540 ± 0.226 | 1 <<< 2 |
| Genetic cluster | BIO12 | C1 | C4 | -0.439 ± 0.248 | 1 < 2 |
| Genetic cluster | BIO12 | C2 | C3 | 0.603 ± 0.219 | 1 >> 2 |
| Genetic cluster | BIO12 | C2 | C4 | 2.514 ± 0.286 | 1 >>> 2 |
| Genetic cluster | BIO12 | C3 | C4 | 1.239 ± 0.248 | 1 >>> 2 |
| Genetic cluster | BIO15 | C1 | C2 | 3.346 ± 0.281 | 1 >>> 2 |
| Genetic cluster | BIO15 | C1 | C3 | -1.337 ± 0.223 | 1 <<< 2 |
| Genetic cluster | BIO15 | C1 | C4 | -4.402 ± 0.328 | 1 <<< 2 |
| Genetic cluster | BIO15 | C2 | C3 | -4.285 ± 0.289 | 1 <<< 2 |
| Genetic cluster | BIO15 | C2 | C4 | -10.234 ± 0.474 | 1 <<< 2 |
| Genetic cluster | BIO15 | C3 | C4 | -2.639 ± 0.272 | 1 <<< 2 |
| Genetic cluster | pH | C1 | C2 | -2.325 ± 0.256 | 1 <<< 2 |
| Genetic cluster | pH | C1 | C3 | -0.102 ± 0.212 | 1 ~ 2 |
| Genetic cluster | pH | C1 | C4 | -0.126 ± 0.247 | 1 ~ 2 |
| Genetic cluster | pH | C2 | C3 | 1.321 ± 0.227 | 1 >>> 2 |
| Genetic cluster | pH | C2 | C4 | 2.626 ± 0.289 | 1 >>> 2 |
| Genetic cluster | pH | C3 | C4 | 0.017 ± 0.240 | 1 ~ 2 |
| Genetic cluster | % Agriculture | C1 | C2 | 1.143 ± 0.234 | 1 >>> 2 |
| Genetic cluster | % Agriculture | C1 | C3 | 1.686 ± 0.229 | 1 >>> 2 |
| Genetic cluster | % Agriculture | C1 | C4 | -2.113 ± 0.272 | 1 <<< 2 |
| Genetic cluster | % Agriculture | C2 | C3 | 0.692 ± 0.220 | 1 >> 2 |
| Genetic cluster | % Agriculture | C2 | C4 | -4.169 ± 0.329 | 1 <<< 2 |
| Genetic cluster | % Agriculture | C3 | C4 | -5.913 ± 0.346 | 1 <<< 2 |
| Genetic cluster | % Urban | C1 | C2 | 3.416 ± 0.282 | 1 >>> 2 |
| Genetic cluster | % Urban | C1 | C3 | 3.966 ± 0.277 | 1 >>> 2 |
| Genetic cluster | % Urban | C1 | C4 | 2.535 ± 0.282 | 1 >>> 2 |
| Genetic cluster | % Urban | C2 | C3 | 0.137 ± 0.217 | 1 ~ 2 |
| Genetic cluster | % Urban | C2 | C4 | -0.321 ± 0.250 | 1 < 2 |
| Genetic cluster | % Urban | C3 | C4 | -0.438 ± 0.241 | 1 < 2 |
| Chlorotype group | BIO1 | A | B | 0.801 ± 0.234 | 1 >>> 2 |
| Chlorotype group | BIO1 | A | C | 4.314 ± 0.341 | 1 >>> 2 |
| Chlorotype group | BIO1 | B | C | 2.271 ± 0.264 | 1 >>> 2 |
| Chlorotype group | BIO2 | A | B | 0.499 ± 0.231 | 1 > 2 |
| Chlorotype group | BIO2 | A | C | 1.408 ± 0.267 | 1 >>> 2 |
| Chlorotype group | BIO2 | B | C | 0.386 ± 0.238 | 1 > 2 |
| Chlorotype group | BIO4 | A | B | 1.983 ± 0.252 | 1 >>> 2 |
| Chlorotype group | BIO4 | A | C | 3.790 ± 0.327 | 1 >>> 2 |
| Chlorotype group | BIO4 | B | C | 1.648 ± 0.253 | 1 >>> 2 |
| Chlorotype group | BIO12 | A | B | -1.894 ± 0.250 | 1 <<< 2 |
| Chlorotype group | BIO12 | A | C | -3.164 ± 0.310 | 1 <<< 2 |
| Chlorotype group | BIO12 | B | C | -2.420 ± 0.268 | 1 <<< 2 |
| Chlorotype group | BIO15 | A | B | 4.057 ± 0.298 | 1 >>> 2 |
| Chlorotype group | BIO15 | A | C | 2.353 ± 0.288 | 1 >>> 2 |
| Chlorotype group | BIO15 | B | C | -1.204 ± 0.246 | 1 <<< 2 |
| Chlorotype group | pH | A | B | -0.836 ± 0.234 | 1 <<< 2 |
| Chlorotype group | pH | A | C | 1.608 ± 0.271 | 1 >>> 2 |
| Chlorotype group | pH | B | C | 2.687 ± 0.273 | 1 >>> 2 |
| Chlorotype group | % Agriculture | A | B | -0.069 ± 0.230 | 1 ~ 2 |
| Chlorotype group | % Agriculture | A | C | 0.443 ± 0.255 | 1 > 2 |
| Chlorotype group | % Agriculture | B | C | 0.736 ± 0.241 | 1 >> 2 |
| Chlorotype group | % Urban | A | B | 3.439 ± 0.284 | 1 >>> 2 |
| Chlorotype group | % Urban | A | C | 2.502 ± 0.292 | 1 >>> 2 |
| Chlorotype group | % Urban | B | C | -0.273 ± 0.238 | 1 < 2 |

**Table S2.** Mean (±SE) values for genetic units and environmental variables included in SDMs and altitude.

| Genetic unit | Altitude  (m.a.s.l.) | BIO1  (°C) | BIO2  (°C) | BIO4  (CV) | BIO12  (mm) | BIO15  (CV) | pH | Agriculture  Land (%) | Urban  Cover (%) |
| --- | --- | --- | --- | --- | --- | --- | --- | --- | --- |
| Non-OVR | 663.1(23.9) | 13.0(0.2) | 11.7(0.1) | 5.84(0.53) | 785.1(20.1) | 40.0(1.0) | 5.6(.01) | 37.0(2.6) | 8.3(1.5) |
| OVR | 1110.8(40.3) | 10.3(0.2) | 12.0(0.1) | 6.02(0.54) | 738.2(30.1) | 33.2(0.9) | 5.9(0.1) | 26.5(3.5) | 1.57(.8) |
| C1 | 784.0(25.5) | 11.7(.01) | 11.8(0.1) | 5.71(0.72) | 772.6(29.9) | 35.5(0.8) | 5.4(0.1) | 40.4(3.3) | 9.6(2.2) |
| C2 | 1019.0(67.1) | 10.4(0.4) | 11.5(0.3) | 5.95(0.73) | 842.6(45.3) | 26.2(1.1) | 6.5(0.1) | 14.3(3.8) | 2.3(1.6) |
| C3 | 965.8(103.3) | 12.3(0.6) | 12.0(0.2) | 6.24(0.98) | 772.9(48.4) | 47.4(2.0) | 5.7(0.1) | 13.6(4.6) | 2.2(1.6) |
| C4 | 470.6(35.6) | 15.9(0.2) | 12.5(0.2) | 6.28(0.68) | 656.5(23.0) | 57.4(1.5) | 5.3(0.1) | 59.4(6.9) | 4.2(3.5) |
| A | 750.7(37.4) | 12.8(0.3) | 12.0(0.1) | 5.97(0.70) | 736.0(27.8) | 41.6(1.2) | 5.6(0.1) | 39.1(3.6) | 8.0(2.2) |
| B | 777.4(51.2) | 11.8(0.3) | 11.6(0.2) | 5.76(0.79) | 801.6(29.3) | 32.2(1.5) | 5.7(0.1) | 34.5(4.6) | 1.8(1.0) |
| C | 1011.8(106.7) | 9.8(0.6) | 11.4(0.3) | 5.23(1.66) | 988.7(78.6) | 31.5(3.1) | 5.4(0.2) | 31.6(8.7) | 3.0(2.6) |

Climatic variables: BIO1, annual mean temperature; BIO2, mean diurnal temperature range; BIO4, temperature seasonality; BIO12, annual precipitation; BIO15, precipitation seasonality. OVR categories: OVR and non-OVR; nuclear genetic clusters: C1, C2, C3 and C4; chloroplast genetic groups: A, B and C.

**Table S3.** Climatic variable percent contribution to the fit of the models.

|  | Species | Phenotypic categories | | Genetic clusters | | | | Chlorotype groups | | |
| --- | --- | --- | --- | --- | --- | --- | --- | --- | --- | --- |
| Variable | – | Non-OVR | OVR | C1 | C2 | C3 | C4 | A | B | C |
| BIO1 | **45.61** | **24.66** | **87.06** | **79.42** | 16.67 | 26.78 | 6.10 | **29.81** | 7.15 | **73.82** |
| BIO2 | 3.79 | 3.34 | 0.80 | 1.69 | 4.70 | 1.50 | 0.11 | 3.34 | 1.85 | 0.00 |
| BIO4 | 5.43 | 5.84 | 10.25 | 8.83 | 7.00 | **29.28** | **30.56** | 7.28 | 3.81 | 18.16 |
| BIO12 | **36.14** | **47.02** | 0.55 | 5.30 | 12.04 | 6.25 | 2.84 | **40.79** | **37.54** | 0.76 |
| BIO15 | 9.03 | 19.15 | 1.37 | 4.76 | **59.60** | **36.19** | **60.43** | 18.78 | **49.64** | 7.25 |

Climatic variables: BIO1, annual mean temperature; BIO2, mean diurnal temperature range; BIO4, temperature seasonality; BIO12, annual precipitation; BIO15, precipitation seasonality. The largest contributions summing more than 50% per genetic unit are given in bold face.
